# A Dynamic Model for Imputing Missing Medical Data: A Multiobjective Particle Swarm Optimization Algorithm

**DOI:** 10.1155/2021/1203726

**Published:** 2021-10-08

**Authors:** Peyman Almasinejad, Amin Golabpour, Mohammad Reza Mollakhalili Meybodi, Kamal Mirzaie, Ahmad Khosravi

**Affiliations:** ^1^Department of Computer Engineering, Maybod Branch, Islamic Azad University, Maybod, Iran; ^2^Shahrood University of Medical Sciences, Shahroud, Iran; ^3^Center for Health Related Social and Behavioral Sciences Research, Shahroud University of Medical Sciences, Shahroud, Iran

## Abstract

Missing data occurs in all research, especially in medical studies. Missing data is the situation in which a part of research data has not been reported. This will result in the incompatibility of the sample and the population and misguided conclusions. Missing data is usual in research, and the extent of it will determine how misinterpreted the conclusions will be. All methods of parameter estimation and prediction models are based on the assumption that the data are complete. Extensive missing data will result in false predictions and increased bias. In the present study, a novel method has been proposed for the imputation of medical missing data. The method determines what algorithm is suitable for the imputation of missing data. To do so, a multiobjective particle swarm optimization algorithm was used. The algorithm imputes the missing data in a way that if a prediction model is applied to the data, both specificity and sensitivity will be optimized. Our proposed model was evaluated using real data of gastric cancer and acute T-cell leukemia (ATLL). First, the model was then used to impute the missing data. Then, the missing data were imputed using deletion, average, expectation maximization, MICE, and missForest methods. Finally, the prediction model was applied for both imputed datasets. The accuracy of the prediction model for the first and the second imputation methods was 0.5 and 16.5, respectively. The novel imputation method was more accurate than similar algorithms like expectation maximization and MICE.

## 1. Introduction

Disease treatment is closely linked to medical observation and data interpretation. Medical data collection and interpretation are the foundation of medical and health care since the data greatly affect decision-making. In fact, all health care measures are linked to medical data collection, interpretation, and application [1].

Missing data are the values that have not been recorded for a variable and are a challenge in preprocessing of data in medical sciences. Missing medical data occurs for different reasons and results in the poor quality of extracted information by data mining [2]. Therefore, imputation and deletion of missing data are necessary approaches for preprocessing and data cleaning to improve the data quality [3–5]. Data deletion will eliminate all the information of the records and results in a low quality of interpretation. However, data imputation with suitable values results in high-quality interpretation and knowledge [3, 6, 7].

In recent years, several methods have been proposed to solve this problem. However, these methods will reduce the quality of medical data since they introduce bias. The majority of the models often improve only accuracy, specificity, or sensitivity and cannot improve all of them simultaneously.

## 2. Background

One of the problems during data collection is missing or not reporting some data for dependent and independent variables. Several mechanisms of missing data exist: (i) missing completely at random (MCAR), (ii) missing at random (MAR), (iii) missing not at random (MNAR), and (iv) nonignorable missingness (NIM). Knowing these mechanisms plays an important role in the selection of a suitable analysis and interpretation method [8, 9].

Little and Robin described missing completely at random (MCAR) as “if the probability of a solution is not linked to neither the observed value nor the missing value that could be collected, the missing value is MCAR” (7). MCAR is defined as follows:

(1) $$\begin{array}{c} p\left(R|Y_{\text{missing}},Y_{\text{observed}},u\right)=p\left(R|u\right). \end{array}$$

In other words, if the probability of missing *Y* is not linked to the observed value of *Y* but linked to undetermined parameters (*u*), these values are MCAR. In this case, the missing values can be replaced with a random subsample of complete data. For example, consider variables *X* and *Y* are age and income, respectively, and there are missing values of income. If the incomes are recorded/missed similarly for all individuals regardless of their age or income, the missing values are MCAR [3, 10].

Missing at random (MAR) assumption is stated as follows:

(2) $$\begin{array}{c} p\left(R|Y_{\text{missing}},Y_{\text{observed}},u\right)=p\left(R|Y_{\text{observed}},u\right), \end{array}$$

which means that the conditional probability of missing *Y*, given both *Y*_missing_ and *Y*_observed_, equals the conditional probability of missed *Y* values given observed *Y* values. In other words, the probability of missing an observation might be related to observed values but not to the missing value itself. For example, consider variables *X* and *Y* are age and income, respectively, and there are missing values of income. If the missed values are observed in a specific age group, the missed values type is MAR. The limitations of MAR are less than that of MCAR. Thus, MCAR is a special MAR [3, 10].

Missing not at random (MNAR) is the type of missed data when the variable itself is the cause of missing data. In the age and income example above, assume that there are missed values only of income. If the missed values are observed in a specific income range, the missed values type is MNAR [3, 11].

Some types of missing data adversely affect the analysis more than other types. Therefore, when encountering missing values, the most important measure is to determine the type of missing data [12, 13].

In this study, a model is proposed based on multiobjective particle swarm optimization and data mining algorithms. The model can improve the specificity, sensitivity, and accuracy of medical data and can be used for both continuous and discrete data.

## 3. Materials and Methods

There are two major methods to impute missing data. (i) The missing data are exactly calculated. This method is not applicable in medical sciences since little imputation error will adversely affect all calculations, and physicians cannot rely on the analyses. (ii) The missing data are imputed based on another parameter such as the population “mean,” which is not related to the missing value itself. This method is applicable in medical sciences [14].

**Figure 1:**
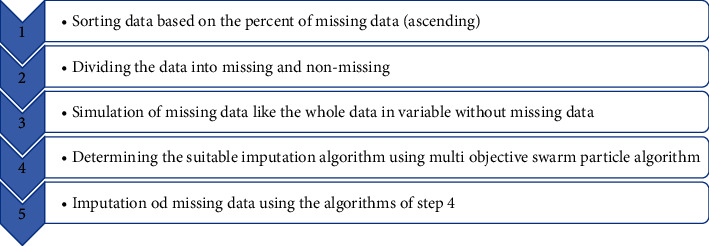
The steps of the proposed model.

The second method has been used in our proposed model to impute missing data. The model has five steps (Figure 1).

**Step 1 .**

The data are sorted ascending. Then, a prediction model is defined based on the number of variables that include missing data as follows:

(3) $$\begin{array}{c} x_{1}={\text{Predict}}_{1}\left(x_{2}\right),\\ x_{2}={\text{Predict}}_{2}\left(x_{1}\right),\\ x_{3}={\text{Predict}}_{3}\left(x_{1},x_{2}\right),\\ x_{4}={\text{Predict}}_{4}\left(x_{1},x_{2},x_{3}\right),\\ \vdots \\ x_{n}={\text{Predict}}_{n}\left(x_{1},x_{2},\ldots ,x_{n-1}\right). \end{array}$$

The missing data of variable *x*_1_ are imputed based on the Predict_1_ model and *x*_2_ variable. Then, the missing data of variable *x*_2_ are imputed based on the Predict_2_ model and *x*_1_ variable. Thus, all missing values of both *x*_1_ and *x*_2_ variables are imputed. Afterward, the missing data of variable *x*_3_ are imputed based on the Predict_3_ model and *x*_1_ and *x*_2_ variables. This process will continue until missing data of the last variable are imputed. To do so, we used a learning system based on a multiobjective particle swarm optimization algorithm to find the best prediction method for imputation. For example, k-nearest neighbors (KNN) prediction model might perform better than other models to predict missing *x*_2_ data using *x*_1_, and the support vector machine (SVM) prediction model might perform better than other methods to predict missing *x*_3_ data using *x*_1_ and *x*_2_. Optimization algorithms are used to determine which model performs better [15].

**Step 2 .**

The data are divided into two groups: (i) records without missing data or observational data and (ii) records with missing data. The minimum number of observational data should be at least 50% of all data. If the number of these records is less than 50%, the records with missing data are imputed using multivariate imputation by chained equations (MICE) to obtain at least 50% data. Then, the algorithms are processed for analysis.

**Step 3 .**

The type of missing data, that is, MCAR, MAR, and MNAR, should be determined. To do so, first using the Little test [16], missing data pattern is determined to be MCAR or not. Second, if the pattern is not MCAR and there is information about the data that determines the type of missing, this type will be the basis of analysis. Otherwise, the pattern is considered MNAR. Finally, the missing data are generated based on the observational data. For example, 30% of variable 1 is missing. Thus, 30% of the variable 1 in the observational data are eliminated based on the type of missing data.

**Figure 2:**
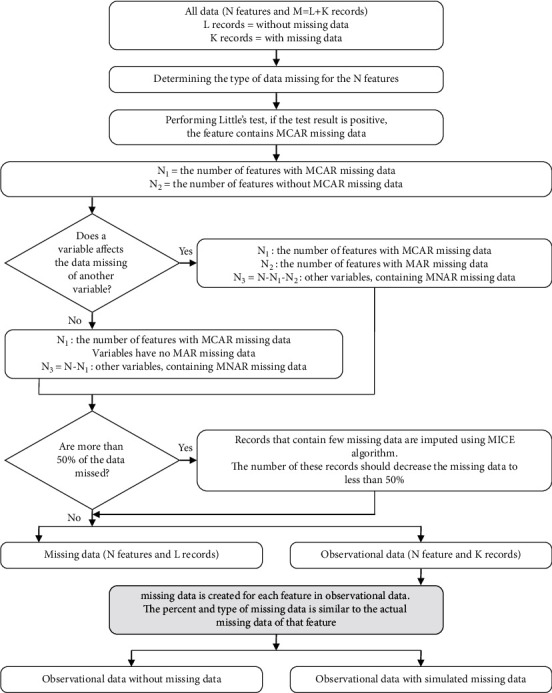
The process of data classification and missing data simulation.

Figure 2 shows the process of dividing the data into observational, missing, and simulated.

**Step 4 .**

The best algorithm should be determined for each prediction (equation (3)) using a multiobjective particle swarm optimization algorithm. After that, the data are categorized as (i) observational data without missing data, (ii) observational data with missing data that are simulated, and (iii) data containing real missing data.

Observational data that do not contain missing data are used to determine the imputation model. Observational data with simulated data are used to evaluate and optimize the model. The algorithm imputes the missing data after optimization. In our model, a prediction model is proposed for any feature that has missing data so that the prediction model can more accurately impute the missing data.

Prediction models are either discrete or continuous. The former is used when the input variables are continuous and discrete independent, and the dependent variable is discrete. The latter is used when the input variables are continuous and discrete independent, and the dependent variable is continuous.  Table 1 shows continuous and discrete algorithms that have been used for our model. Note that combinatory algorithms can be used to strengthen the model.

The best approach is to evaluate all possible data imputation methods and then determine the best imputation algorithm for each feature.

## 4. Problem-Solving Using a Multiobjective Particle Swarm Optimization Algorithm

In the proposed model, the multiobjective particle swarm optimization algorithm [17] finds the best imputation algorithm for the missing data of each feature. Depending on the feature, the continuous/discrete algorithm can be used.

### 4.1. Particle Structure

**Figure 3:**
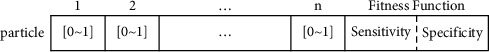
The structure of the proposed particle.

Each particle indicated the algorithms that are used in the proposed model. The abscissa of a particle is the number of independent variables that contain missing data, and the values of a particle are continuous between 0 and 1. Since we need to choose an algorithm based on the row number of algorithms in the algorithm table, we face a discrete condition. Thus, the continuous interval [0, 1] should be transformed to the discrete interval [1, *n*]. equation (4) shows this transformation. In fact, a multiobjective particle swarm optimization algorithm is transformed from continuous interval to discrete interval using the “*f*  ” function (equation (5)). “*n*” is 18 and 9 for continuous and discrete variables, respectively. The “*f*  ” function gives an integer. For example, if the result of the first index of the particle, whose variable is discrete, is 14 (based on equation (6)), a 9-NN algorithm should be used to predict the first missing data-containing feature. Likewise, if the result of the second index of the particle, whose variable is continuous, is 6 (based on equation (5)), a 6-NN algorithm should be used to predict the first missing data-containing feature.  Figure 3 shows the structure of the multiobjective particle swarm optimization algorithm. The particle abscissa is “*n*,” which is the number of features that contain missing data, and “*n*” is a continuous parameter between 0 and 1.

(4) $$\begin{array}{c} \left[0,1\right]\overset{f}{⟶}\left[1,n\right], \end{array}$$

(5) $$\begin{array}{c} x_{i}=\left[0,1\right], \end{array}$$

### 4.2. Generation of the First Population

First, 100 particles are generated using uniform distribution as follows [18]; a number between 0 and 1 is randomly assigned to each one:

(6) $$\begin{array}{c} \text{particle}=U\left(0,1\right). \end{array}$$

### 4.3. Fitness Function

**Figure 4:**
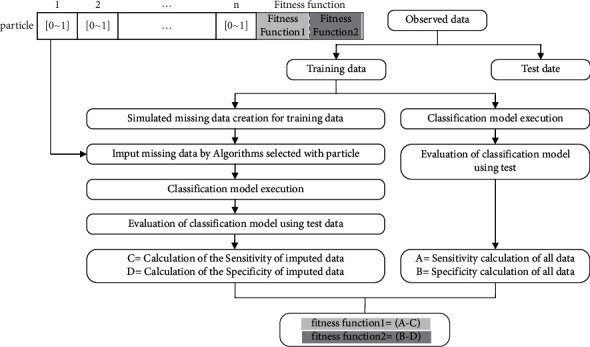
The proposed fitness function.

The fitness function of each particle determines how suitable an imputation algorithm is for the corresponding feature. First, the observational data are classified, and the accuracy of the model is evaluated. Then, missing data are intentionally created in the observational data (Figure 1). Afterward, the created missing data are imputed using the determined algorithms. The data are again classified, and the sensitivity and accuracy of the model are evaluated. Finally, the difference of sensitivity and specificity criteria between the two model evaluation modes, including the use of complete data and data with artificial missing values, are calculated. This process is repeated 100 times, and the average of the differences is considered as the fitness function. The lower this value, the more suitable the particle.  Figure 4 shows the structure of the fitness function.

### 4.4. Velocity Prevention

One of the important aspects of determining the accuracy of an optimization algorithm is to “Explore” and “Exploit” features. “Explore” is the ability of an algorithm to search for the optimized value. “Exploit” is the ability to perform a focused search around a probable area to find the best solution. Thus, an optimized solution is created between these two opposing factors by updating the velocity of PSO as follows [19, 20]:

(7) $$\begin{array}{c} v_{ij}\left(t+1\right)=v_{ij}\left(t\right)+c_{1}r_{1j}\left(t\right)\left[y_{ij}\left(t\right)-x_{ij}\left(t\right)\right]+c_{2}r_{2j}\left(t\right)\left[{\tilde{y}}_{j}\left(t\right)-x_{ij}\left(t\right)\right],\\ v_{ij}\left(t+1\right)=\left\{\begin{array}{cc} v_{ij}\left(t+1\right), & \text{if }v_{ij}\left(t+1\right)<v_{\max,j},\\ v_{\max,j}, & \text{if }v_{ij}\left(t+1\right)\geq v_{\max,j}, \end{array}\right. \end{array}$$

where *v*_max,*j*_ is the maximum speed of the particles in the number of tables and columns. This parameter is important since the search speed is limited by this parameter. If *v*_max,*j*_ is a big number, the Explore capability of the algorithm is increased. On the contrary, if *v*_max,*j*_ is small, the Exploit capability is enhanced. If *v*_max,*j*_ is too small, the swarm might not be able to search the local optima. In addition, the swarm might be entrapped in local optima, and the algorithm cannot exit this point. Large values of *v*_max,*j*_ increase the chance of losing optimal regions. The swarms might jump the optimized solutions and search non-optimal regions. Thus, large *v*_max,*j*_ results in the distancing of the algorithm from optimal regions [19].

*v*_max,*j*_ should be calculated to create a balance and is done as follows:

1. Fast or slow movement
2. Explore and exploit capabilities

*v*_max,*j*_ is considered as a fraction of each dimension as follows:

(8) $$\begin{array}{c} v_{\max,j}=\delta \left(x_{\max,j}-x_{\min,j}\right). \end{array}$$

At first, *δ* equals 1; its value changes in each generation based on the following equation. Note that, *δ* in each generation is 90% less than the previous generation.

(9) $$\begin{array}{c} \delta =0.9^{i},\;i=\text{ineration number}. \end{array}$$

### 4.5. Termination

The algorithm termination depends on the swarm diameter, where normalized diameter approaches 0. *s* is the diameter of the primary swarm space, and *R*_max_ is the maximum diameter, calculated using the following equations [21]:

(10) $$\begin{array}{c} R_{\text{norm}}=\frac{R_{\max}}{\text{diameter}\left(S\right)}, \end{array}$$

(11) $$\begin{array}{c} R_{\max}=x_{m}-\hat{y},\;m=1,\ldots ,n_{s}. \end{array}$$

### 4.6. Final Output

Since the algorithm is multitarget and its output is a set of values, the particle with the highest accuracy is selected. To do so, all final outputs of the algorithm are calculated, and the highest accuracy one is selected. If several outputs with identical accuracies are generated, one is randomly selected.

**Step 5 .**

After the multiobjective particle swarm optimization algorithm determines the imputation algorithm, each algorithm is run according to the described steps until all missing data are imputed.

## 5. Evaluation

The proposed algorithm was evaluated using data of gastric cancer and adult T-cell leukemia/lymphoma (ATLL) patients. Both data sets include missing data in a way that cannot be classified. Thus, the missing data should be imputed.

### 5.1. Imputation of Gastric Cancer Data Based on the Proposed Algorithm

Gastric cancer is one of the most prevalent and life-threatening cancers. It is also more prevalent in males than in females. Tens of thousands of individuals are affected by the disease annually in Iran. The research was a survival study, and 277 individuals, who were admitted to Jahad Daneshgahi Research Center from 2008 to 2015, were included. The data of 197 out of 277 admitted patients were excluded since there was no survival data. Thus, the data set contains data of 80 gastric cancer patients. There are 15 independent and 1 dependent variables.  Table 2 shows the type of the variables. There were missing data in 8 variables from 5% to 88%, and the overall missing data was 29.5% (Table 3).

**Figure 5:**
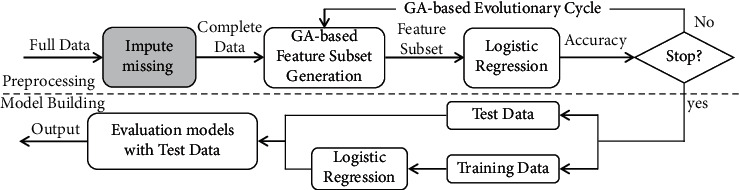
The structure of model design for the prediction of gastric cancer survival time.

**Figure 6:**
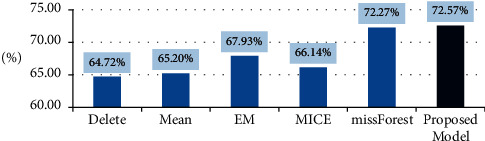
The structure of model design for the prediction of gastric cancer survival time.

Variables with more than 50% missing data were excluded. We imputed the missing data using our proposed model as well as five other imputation algorithms including deletion, average, EM, missFrost, and multivariate imputation by chained equations (MICE). Genetic and logistic regression algorithms were used to design the proposed model [22, 23] using MATLAB software to predict the survival time.  Figure 5 shows the structure of the model. A tenfold method was used to divide the data into training and test groups in all six imputation methods. Both models were performed 10,000 times, and the accuracy was calculated. The mean accuracy was considered the accuracy of the model.  Figure 6 shows the result of all imputation methods [24]. The accuracy of the proposed model was 72.57%, which is the highest.

### 5.2. Imputation of ATLL Data Based on the Proposed Algorithm

ATLL is an advanced malignancy of adults' T cells and is the result of HTLV-1 infection [25, 26]. Twenty-five ATLL patients, who were admitted to Jahad Daneshgahi Research Center from 2016 to 2018, were included. There were 35 independent variables and 1 dependent variable, median overall survival time (Table 4). Twelve independent variables contained missing data, among which fasting blood glucose (FBS) had the highest missing data (48%). Overall, there were 18.47% missing data. Variables with more than 50% missing data were excluded from the study. We imputed the missing data using our proposed model as well as 5 other imputation algorithms including deletion, average, EM, missFrost, and multivariate imputation by chained equations. Genetic and logistic regression algorithms were used to design the proposed model using MATLAB software to predict the survival time. The 30-70 method was used to divide the data into training and test groups in all six methods. The model was performed 10,000 times, and mean accuracy was considered as the accuracy of the models.  Table 5 shows the structure of the model. As shown, the proposed model performed better than other methods, that is, it improved the accuracy by 16.52% compared to other imputation methods.

## 6. Discussion

Missing data have been gained attention in various statistical analyses. Most researchers encounter missing data during data analysis. Several reasons result in missing data. For example, when a researcher uses a questionnaire, the participants might not be willing to answer some questions because of a lack of time or personal questions. Thus, researchers need to properly impute the missing data to be able to analyse the data.

Low-quality data result in the low quality of conclusions. Thus, preprocessing and data cleaning are applied to improve the quality of the data. In case of missing data, one needs to impute the missing data using a suitable method before modelling [5, 27]. Data are missed due to various reasons, and researchers must determine the type of missing data [2, 28, 29]. The reason is that the selection of the method of imputation is different based on the type of missing data. There are three types of missing data: (i) MCAR, which does not depend on other variables, (ii) MAR, which depends on the status of observational data, and (iii) MNAR, which depends on the status of the missing data. We have shown that the type of missing data affects the accuracy of the imputation algorithms.

Enders has stated that if missing data is MCAR, the missing data can be excluded [30]. However, we showed that excluding MCAR missing data decreases the accuracy of classification. In fact, our model selects the best imputation algorithm for a specific type of missing data. We used 18 and 9 variable classification algorithms for discrete and continuous variables, respectively. Then, a training algorithm determines the best algorithm. The training model was performed using a multiobjective particle swarm optimization algorithm. To improve the model, the fitness function was adjusted based on sensitivity and specificity.

To assess the model, the data sets of gastric cancer and ATLL patients were used. In gastric cancer data, the survival time was predicted by the model. The data contained 29.5% missing data, which were imputed by the model. The result indicated that the proposed model improved the accuracy by 6.43% compared to multivariate imputation by chained equations. In ATLL data also, the survival time was predicted by the model. The data contained 18.47% missing data. The result indicated that the proposed model improved the accuracy by 16.52% compared to EM.

The proposed model has several advantages over other methods: (i) in the proposed model, missing data simulation is based on the part of data, which are not missing. Thus, the algorithm uses the same structure for the missing data imputation as the non-missing data. (ii) Most algorithms use a single imputation method to impute missing data. The proposed model is flexible, that is, it determines the best imputation algorithm for the missing data based on the type of Missing data. The proposed model, however, has several disadvantages: (i) it is slow due to the multiobjective particle swarm optimization algorithm and (ii) it depends on the variables rather than the records. Thus, we suggest developing a dynamic algorithm that imputes the missing data based on the records.

## Figures and Tables

**Table 1:** The continuous and discrete algorithms that were used in the proposed model.

No.	Algorithm name
Discrete algorithms	
1	Support vector machines-linear
2	Support vector machines-quadratic
3	Support vector machines-polynomial
4	Support vector machines-RBF = 5
5	Support vector machines-RBF = 2
6	Support vector machines-RBF = 1
7	Support vector machines-RBF = 0.5
8	Support vector machines-RBF = 0.2
9	Support vector machines-RBF = 0.1
10	1-NN
11	3-NN
12	5-NN
13	7-NN
14	9-NN
15	Decision tree (C4.5)
16	Artificial neural network feed forward
17	Logistic regression
18	Naïve Bayesian

Continuous algorithms	
1	Support vector regression (SVR)
2	1-NN
3	3-NN
4	5-NN
5	7-NN
6	9-NN
7	Continuous decision tree (CART)
8	Artificial neural network feed forward
9	Multiple regression

**Table 2:** The characteristics of gastric cancer variables.

ID	Variable name	Variable type	Notes
1	Sex	Nominal	61 males and 19 females
2	Birth year	Interval	Minimum = 1,305, maximum = 1,346
3	Education	Ordinal	(1) Illiterate, (2) underdiploma
4	Race	Ordinal	(1) Fars, (2) Kurd, (3) Turk
5	PMH	Ordinal	(1) Hypertension (HTN), (2) coronary artery disease (CAD), (3) diabetes mellitus (DM), (4) DM + HTN, (5) DM + HTN + CAD, (6) HTN + CAD
6	Age at diagnosis	Interval	Minimum = 46, maximum = 87
7	FH of gastric cancer	Ordinal	Family history of gastric cancer: (1) first-degree relative (FDR), (2) second-degree relatives (SDR)
8	Age at dx of family GC	Interval	Family's age at diagnosis: minimum = 45, maximum = 82
9	Hx of other GI cancer	Ordinal	History of other GI cancer (1) First-degree relative (2) Second-degree relatives
10	Types of other GI cancer	Ordinal	(1) Small intestine, (2) liver, (3) esophagus, (4) large intestine
11	Hx of non-GI cancer	Ordinal	(1) First-degree relative (2) Second-degree relatives
12	Treatment	Ordinal	(1) Surgery, (2) surgery + chemo + radio, (3) chemo
13	Cause of death	Ordinal	(1) cancer, (2) MI, (3) PTE
14	Pathology	Ordinal	(1) Adenocarcinoma, (2) inflammatory tumour, (3) mucinous adenocarcinoma, (4) neuroendocrine carcinoma, (5) signet ring cell carcinoma, (6) GIST tumour, (7) undifferentiated carcinoma
15	Addiction	Nominal	17 subjects: addicted, 63 subjects: non-addicted
16	Survival	Nominal	33 and 67 subjects pass away after one and two years, respectively

**Table 3:** The percent of missing data in independent variables of gastric cancer data.

ID	Variable name	Missing		Valid *N*
		*N*	Percent	
1	Hx of non-GI cancer	71	88.75	9
2	Type of other GI cancer	64	80.00	16
3	Hx of other GI cancer	64	80.00	16
4	Age at Dx of family GC	58	72.50	22
5	FH of gastric cancer	57	71.25	23
6	PMH	35	43.75	45
7	Age at diagnosis	4	5.00	76
8	Birth year	1	1.25	79

**Table 4:** The number and percent of missing data of independent variables.

ID	Variable name	Missing		Valid *N*
		*N*	Percent	
1	FBS	12	48.0	13
2	Rb	8	32.0	17
3	P53	8	32.0	17
4	CDK4	8	32.0	17
5	CDK2	8	32.0	17
6	Creat	5	20.0	20
7	Urea	5	20.0	20
8	CA	5	20.0	20
9	MCV	1	4.0	24
10	MCHC	1	4.0	24
11	MCH	1	4.0	24
12	RBC	1	4.0	24

**Table 5:** The comparison of the proposed model of imputation with EM algorithm for ATLL patients' data.

Algorithm name	Sensitivity (%)	Specificity (%)	Accuracy (%)	PPV^+^ (%)	PPV^−^ (%)	*F*-measure (%)
Delete missing	47.00	40.60	45.95	47.1	38.95	45.47
Mean algorithm	47.37	51.20	53.77	44.77	49.83	43.49
Expectation maximization	62.57	69.25	70.23	64.44	65.35	61.31
MICE algorithm	46.16	49.28	53.37	45.92	46.88	43.09
missForest algorithm	58.30	62.65	64.65	58.15	61.00	56.09
Proposed algorithm	86.15	82.4	86.75	83.57	84.67	83.50

## Data Availability

The data that support the findings of this study are available from the corresponding author upon reasonable request.
