# Corrigendum to “A Dynamic Model for Imputing Missing Medical Data: A Multiobjective Particle Swarm Optimization Algorithm”

**DOI:** 10.1155/2022/9861057

**Published:** 2022-07-21

**Authors:** Peyman Almasinejad, Amin Golabpour, Mohammad Reza Mollakhalili Meybodi, Kamal Mirzaie, Ahmad Khosravi

**Affiliations:** ^1^Department of Computer Engineering, Maybod Branch, Islamic Azad University, Maybod, Iran; ^2^Shahroud University of Medical Sciences, Shahroud, Iran; ^3^Center for Health Related Social and Behavioral Sciences Research, Shahroud University of Medical Sciences, Shahroud, Iran

In the article titled “A Dynamic Model for Imputing Missing Medical Data: A Multiobjective Particle Swarm Optimization Algorithm” [1], there was a spelling error in affiliation two. The correct affiliation is “Shahroud University of Medical Sciences, Shahroud, Iran” and it is shown above.
